# High-Temperature Measurement of a Fiber Probe Sensor Based on the Michelson Interferometer

**DOI:** 10.3390/s22010289

**Published:** 2021-12-31

**Authors:** Jiahao Guo, Siping Lian, Ying Zhang, Yufeng Zhang, Dezhi Liang, Yongqin Yu, Ruohang Chen, Chenlin Du, Shuangchen Ruan

**Affiliations:** 1 College of Physical Science and Technology, Guangxi Normal University, Guilin 541004, China; guojiahao18@163.com (J.G.); 18824386925@163.com (S.L.); 2 Key Laboratory of Advanced Optical Precision Manufacturing Technology of Guangdong Higher Education Institutes, Shenzhen Technology University, Shenzhen 518060, China; zhangying@sztu.edu.cn (Y.Z.); zhangyufeng@sztu.edu.cn (Y.Z.); liangdezhi@sztu.edu.cn (D.L.); duchenlin@sztu.edu.cn (C.D.); ruanshuangchen@sztu.edu.cn (S.R.)

**Keywords:** high temperature sensor, fiber probe sensor, Michelson Interferometer

## Abstract

In this paper, a fiber probe high-temperature sensor based on the Michelson Interferometer (MI) is proposed and experimentally verified. We used a fiber splicing machine to fabricate a taper of the single-mode fiber (SMF) end. The high order modes were excited at the taper, so that different modes were transmitted forward in the fiber and reflected by the end face of the fiber and then recoupled back to the fiber core to form MI. For comparison, we also coated a thin gold film on the fiber end to improve the reflectivity, and the reflection intensity was improved by 16 dB. The experimental results showed that the temperature sensitivity at 1506 nm was 80 pm/°C (100 °C~450 °C) and 109 pm/°C (450 °C~900 °C). The repeated heating and cooling processes showed that the MI structure had good stability at a temperature up to 900 °C. This fiber probe sensor has the advantages of a small size, simple structure, easy manufacturing, good stability, and broad application prospects in industrial and other environments.

## 1. Introduction

Optical fiber sensors have the advantages of a small size, light weight, antielectromagnetic interference, anticorrosion, high sensitivity, and can measure pressure, temperature, stress (strain), magnetic fields, the refractive index, and various physical quantities [1,2,3,4,5,6,7,8,9,10]. Among them, temperature measurement plays an important role in industry, mechanical processing, ore exploration, mining and oil and gas storage, as well as medicine; however, the traditional thermocouple temperature sensor has certain security risks in these environments, so the optical fiber temperature sensor has developed rapidly in recent years [11,12]. In industries such as metallurgy, electric power, building materials, and the chemical industry, there are more and more situations where the working environment temperature exceeds 500 °C, that is, the demand for monitoring the high-temperature environment is also increasing. The common fiber temperature sensors are Fiber Bragg grating (FBG) and long period fiber grating (LPFG) [13,14,15]. For example, as early as 2004, D Grobnic et al. proposed a high-temperature stable Bragg grating that has been inscribed in sapphire fibers and has been tested as a sensor for temperatures up to 1700 °C [16]. However, sapphire fibers and femtosecond laser lithography undoubtedly increase the cost and difficulty of sensor fabrication. In 2020, Zhou G R et al. used a CO_2_ laser to fabricate an LPFG on a single-mode fiber (SMF) that works from room temperature to 875 °C [17]. Although the LPFG does not use a special fiber, it requires laser lithography. The temperature sensor made of a single crystal sapphire fiber can work at 1800 °C, which is a very good high-temperature sensor, but it is relatively expensive. At the same time, a single crystal sapphire fiber without a cladding structure will react with the surrounding environment at high temperature, affecting the high-temperature transmission performance of the fiber [18,19,20,21,22]. The fiber Fabry–Perot (F-P) sensor also provides a method for high-temperature measurement. It can work stably in the high-temperature range. At present, the preparation methods of F–P mainly include wet chemical etching [23], arc discharge [24], laser [25], and polymer-assisted methods [26]. These methods increase the preparation cost of F–P sensors [27]. Fiber optic Mach–Zehnder interferometer (MZI) sensors can also be used to achieve high-temperature sensing, but often require a variety of special structures of optical fibers spliced together [28,29].

In 2009, Tian Z B and others designed and fabricated an MI with an abrupt tapered structure on an SMF to measure the refractive index [30]. In 2015, Fu H W et al. proposed the use of the SMF and a small section of a multi-mode fiber (MMF) to be fusion spliced, which widened the splice point to form a convex lumbar structure and added a biofilm and silver film on the end surface to form an MI [31]. In 2015, Zhao N et al. assembled a thin-core fiber (TCF) at one end of the SMF to form a cone-shaped MI. The temperature sensitivity of the MI interferometer is 0.14 nm/°C in the range of 30 °C~800 °C [32]. In 2018, Zhao Y J et al. proposed and demonstrated a multi-parameter sensing-integrated fiber MI based on the cascade of an asymmetric twin-core fiber and side-hole fiber (SHF). The temperature sensitivity of the sensor was 10.37 pm/°C [33]. In 2021, Han Y et al. proposed an ultra-short high-temperature fiber sensor based on SMF and a silicon microcap fabricated by simple splicing technology. The sensor has 10.87 pm/°C in the 100~500 °C range and 11.36 pm/°C in the 500~900 °C range [34].

Tian Z B proposed that the SMF was heated and suddenly stretched to a small diameter to form a taper, so that the evanescent field was partially extended from the core to the cladding [30]; they tested the performance of the sensor for refractive index measurement, but did not conduct in-depth research on the temperature characteristics of the sensor. Zhao N proposed that a fiber bi-cone is fabricated by submitting two fibers to high-intensity discharge and large-pushing distance [32]; this sensor has good working performance in a high-temperature environment; however, a special optical fiber was used in the fabrication of this sensor, which increases the complexity of the sensor structure and the production cost. The sensor presented in this paper does not need two sections of fibers splices, with pushing or stretching to form a taper or heat SMF and sudden stretching to a small diameter, extending the evanescent field partially from the core to the cladding. The sensor presented in this paper does not require the splicing of two sections of fibers; it uses only one section of the SMF to form a taper on the optical fiber splicing machine. The sensor structure is very simple and has a low cost to fabricate. After the temperature response test, the results showed that it has high-temperature sensitivity and good stability and repeatability, as well as good application prospects in high-temperature measurements.

## 2. Operation Principle and Device Fabrication

**Figure 1:**
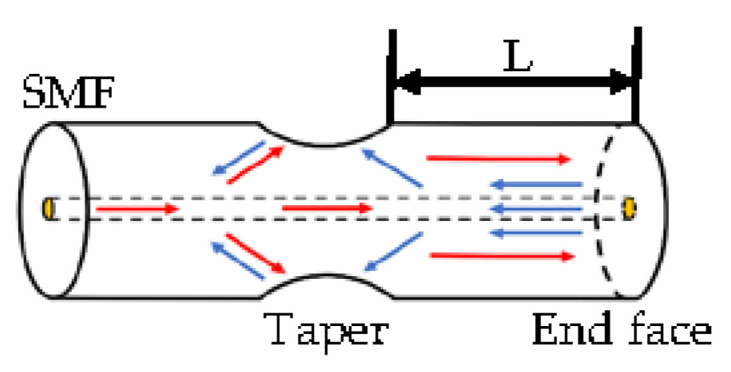
Sketch of the sensor structure and light propagation.

The diagram of the sensor structure and the light propagation in the fiber are shown in Figure 1. When the light propagates in SMF, the high-order modes are excited at the taper, so different modes are transmitted forward in the fiber, reflected by the end face of the fiber, and then recoupled back to the fiber core at the taper to form MI. Its interference can be expressed by the general formula:

(1) $$I=I_{1}+I_{2}+2\sqrt{I_{1}I_{2}}\cos\Phi^{m}$$

where $I_{1}$ and $I_{2}$ represent the light intensity of the respective core mode and high-order cladding mode transmitted in the SMF. $\Phi^{m}$ is the phase difference between the core mode and the high-order mode, and external physical quantities will cause it to change. The expression is:

(2) $$\Phi^{m}=\frac{2\pi \left(n_{eff}^{co}-n_{eff}^{cl}\right)2L}{\lambda }$$

$n_{eff}^{co}$ and $n_{eff}^{cl}$ represent the effective refractive index of the core mode and the cladding mode, respectively, L represents the distance from the taper to the end face of the fiber, λ represents the input light wavelength, and m represents the m-order cladding mode.

We can obtain the interference dip wavelength expression as:

(3) $$\;\;\;\;\;\;\lambda =\frac{2\pi \left(n_{eff}^{co}-n_{eff}^{cl}\right)2L}{\left(2m+1\right)\pi }$$

λ can change with the change in external parameters, so it can be used as a sensing signal to make sensors.

**Figure 2:**
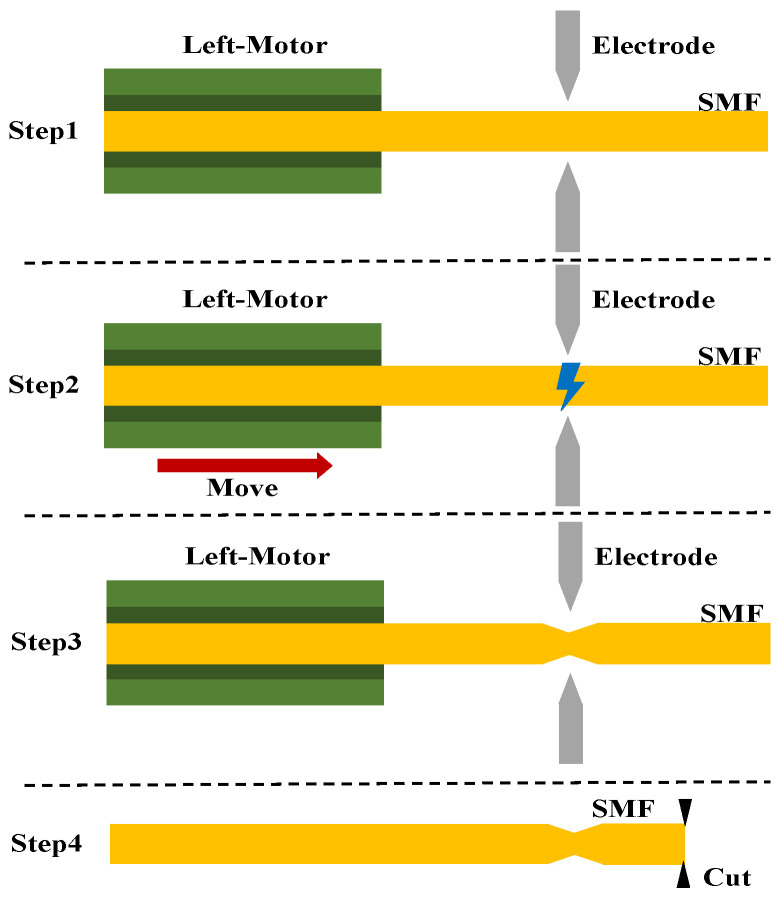
Schematic diagram of the sensor fabrication method.

We use a conventional fiber splicing machine (FITEL s179) to form the taper in SMF. Specific steps are shown in Figure 2. We edited the parameters of the fiber splicer: the discharge intensity was 200, the discharge time was 1500 ms, and the left motor propulsion distance was 15 μm. One section of the SMF was placed in the fiber splicing machine after cleaning; the end face of the fiber exceeded the discharge electrode, and then discharge was performed to construct the taper structure; we did not place anything on the right fixture of the fiber splicer, i.e., only the left fiber formed the taper structure. The light from the superluminescent light-emitting diode (SLED ranging from 1250 nm to 1650 nm) was transmitted through a circulator with sensors and an optical spectrum analyzer (OSA, YOKOGAWA AQ6370B), and the transmission spectra were recorded by an OSA with a resolution of 0.5 nm; the reflection spectrum of the SMF was observed in real time. In this process, OSA records the original reflection spectrum of SLED in Trace A acting as a substrate and records the reflection spectrum of the SMF in Trace B and subtracts it from Trace A. The spectrum thus obtained eliminates the effect of uneven power distribution at different wavelengths of the light source. Then, we moved the fiber into the optical fiber cleaver under the microscope. The fiber was fixed on a three-dimension translational stage by a fiber holder and could be transferred back and forth. Therefore, the distance (L) from the taper to the end face can be controlled.

**Figure 3:**
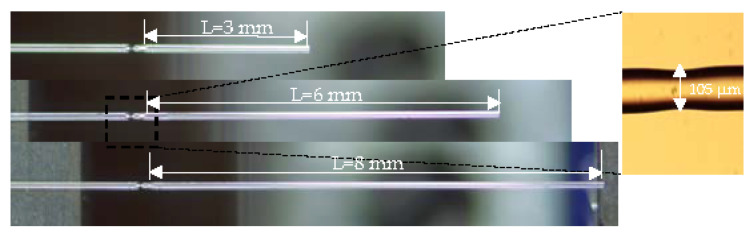
Microscope image of the taper structure of MI with different lengths and enlarged view of the taper.

Figure 3 shows the morphology of the taper with different lengths (L) under the microscope. The distance L from the taper to the end face is controlled to be 3 mm, 6 mm, and 8 mm, and an enlarged view of the taper area was obtained under a microscope (Olympus BX53) to measure the taper size. The taper was obtained with a length of 324 μm and a diameter of 105 μm.

**Figure 4:**
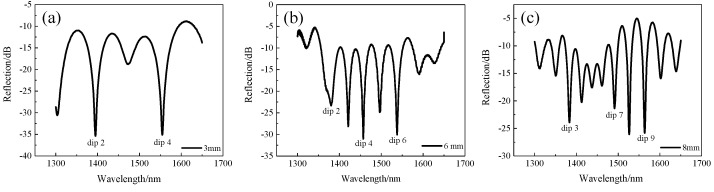
Reflection spectra of the MI structure with different lengths (L). (**a**) L = 3 mm; (**b**) L = 6 mm; (**c**) L = 8 mm.

Their spectra had good contrast as shown in Figure 4a–c. With the increase in the length, the free spectral range (FSR) of the spectrum decreased and more interference peaks appeared.

**Figure 5:**
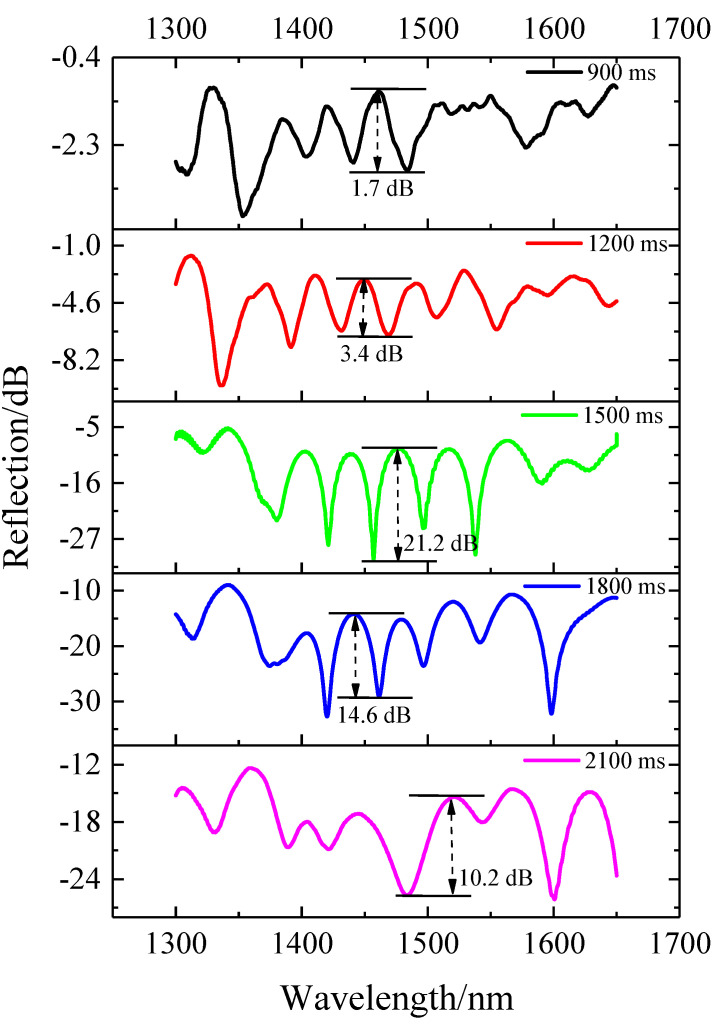
Reflection spectra of the MI structure under different discharge times with L = 6 mm.

Regarding the fabrication of the taper structure, the impact of the discharge time was evaluated. The reflection spectra of the structures with different discharge times are presented in Figure 5. When the electrode discharged the fiber, the high-order modes were excited to interfere with the fundamental mode in the fiber. With the increase in the discharge time, the intensity of the excited high-order mode increased and gradually approached the intensity of the fundamental mode, leading to an increase in the contrast ratio. The reflected spectrum had the best extinction ratio of 21.2 dB when the discharge time was 1500 ms. With the further increase in the discharge time, the contrast decreased and the reflection intensity was also further reduced. Therefore, we used the discharge time of 1500 ms in our experiments.

**Figure 6:**
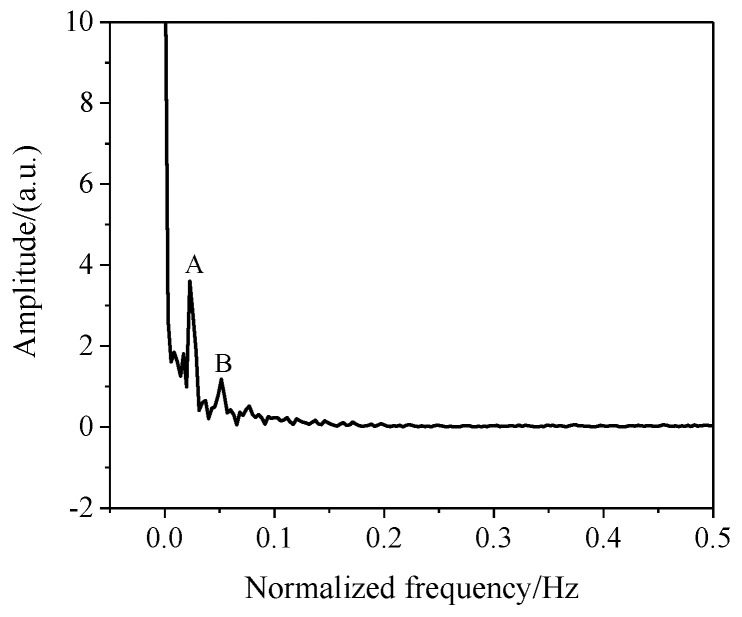
The Fast Fourier Transform curve.

We also analyzed the optical modes transmitted in the sensor. The spectrum of the sensor (1500 ms) in Figure 5 was transformed by Fast Fourier Transform (FFT) in Figure 6, and we observed two obvious independent peaks marked A and B in Figure 6; the corresponding values were 0.02285${\;\mathrm{nm}}^{-1}$ and 0.051423${\;\mathrm{nm}}^{-1}$, rexpectively. Using the reciprocal relationship, we calculated the corresponding FSR values to be 43.75 nm and 19.447 nm.

According to Formula (3), we can obtain FSR [29]:

(4) $$\mathrm{FSR}=\frac{\lambda_{1}\lambda_{2}}{\Delta n_{\mathrm{eff}}\times 2L}$$

$\lambda_{1},\lambda_{2}$ is the wavelength value of two adjacent dips,$\;\Delta n_{\mathrm{eff}}=n_{\mathrm{eff}}^{\mathrm{co}}-n_{\mathrm{eff}}^{\mathrm{cl}}$. We substituted the calculated FSR values at 43.75 nm and 19.447 nm into Formula (4) and calculated that $\Delta n_{\mathrm{eff}}$ was $4.04\times 10^{-3}$ and $9.1\times 10^{-3}$, respectively. Then, these two values were introduced into Formula (5) with the cutoff frequency V, and the cutoff frequencies corresponding to peaks A and B were 3.693 and 5.743.

(5) $$V=\frac{2\pi a}{\lambda_{1}}\sqrt{n_{\mathrm{eff}}^{\mathrm{co}}{}^{2}-n_{\mathrm{eff}}^{\mathrm{cl}}{}^{2}}=\frac{2\pi a}{\lambda_{1}}\sqrt{2n_{\mathrm{eff}}^{\mathrm{co}}\times \Delta n_{\mathrm{eff}}}$$

Table 1 shows the U values of the cut-off and away cut-off for the LP mode: $U_{0}$, $U_{\infty }$. The cut-off indicates that the light waves transmitted in the fiber were at the threshold of the total reflection at the core and cladding interfaces. The away cut-off means that the light wave in the core traveled in a direction close to the fiber axis and always satisfied the total reflection condition. In the same fiber, the different modes have different transmission characteristics, resulting in different U values. We can determine which mode is excited by $U_{0}<$ V $<U_{\infty }$ [35]. According to the above calculation results, we observed that in the sensing structure, the ${\mathrm{LP}}_{11}$ and ${\mathrm{LP}}_{12}$ modes were excited at the taper, so that different modes propagated forward in the fiber and reflected through the fiber end to form the MI and spectra.

After that, the waveguide and dispersion characteristics of the SMF were analyzed by the finite element method (FEM). We used a cladding diameter of 125 µm and a core diameter of 8.6 µm to construct the cross-section of the model and divided the cross-sectional region with a triangular mesh with the boundary conditions set to perfect boundary conditions. Parametric scanning simulations were performed in the range of 1300 nm~1650 nm.

**Figure 7:**
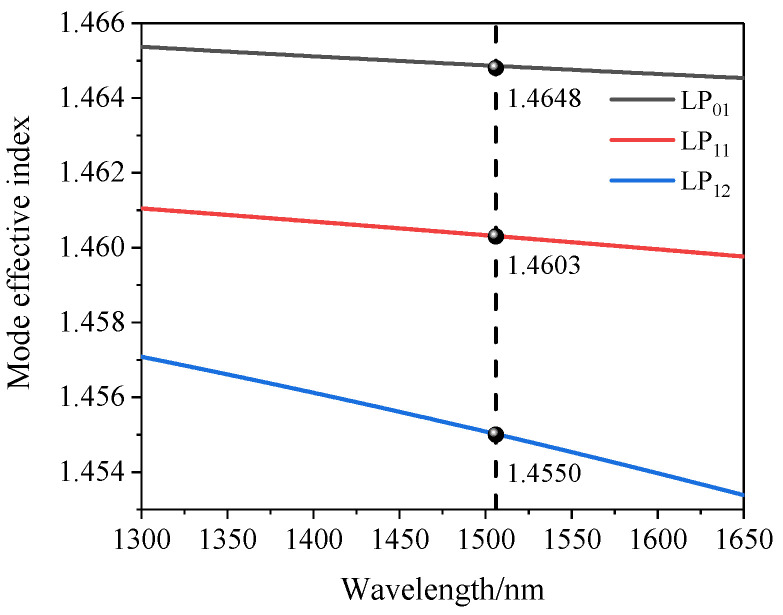
Mode dispersion curve simulated by FEM.

**Figure 8:**
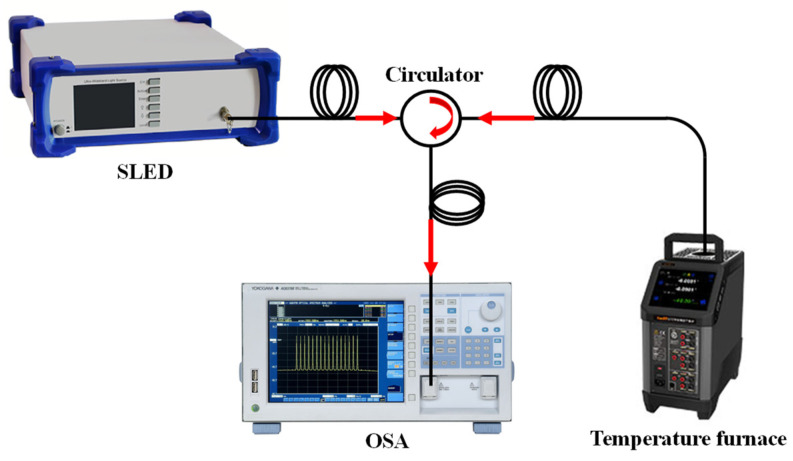
Schematic diagram of the fiber-optic temperature test system.

Then, the mode dispersion curve of this sensing structure was obtained by simulation with FEM, as shown in Figure 7. The simulation results showed the dispersion curves of the basic mode ${\mathrm{LP}}_{01}$ and high-order modes ${\mathrm{LP}}_{11}$ and ${\mathrm{LP}}_{12};$ we calculated the effective mode refractive index values of the three curves at 1506 nm in Figure 8 as 1.4648, 1.4603, and 1.4550. The difference between the effective refractive index of ${\mathrm{the}\;\mathrm{LP}}_{01}$ and ${\mathrm{LP}}_{11}$ modes was $4.4\times 10^{-3}$, and the difference of the effective refractive index of the ${\mathrm{LP}}_{01}$ and ${\mathrm{LP}}_{12}$ modes was $9.8\times 10^{-3}$. These values are similar to the previous calculated values in FFT, so we believe that the reflection spectra of the MI sensor were formed by the interference of ${\mathrm{LP}}_{01}$ with ${\mathrm{LP}}_{11}$ and ${\mathrm{LP}}_{12}$.

## 3. Experiments and Discussion

The experimental setup for investigating the temperature response of this sensing structure is shown in Figure 8. The resolution was 0.1 °C as the temperature varied provided by a temperature furnace (100 °C to 1200 °C). Firstly, a quartz tube was fixed above the temperature furnace, and then the sensor (L = 6 mm) was inserted into the temperature furnace through the quartz tube to ensure that the sensor did not touch the temperature furnace.

**Figure 9:**
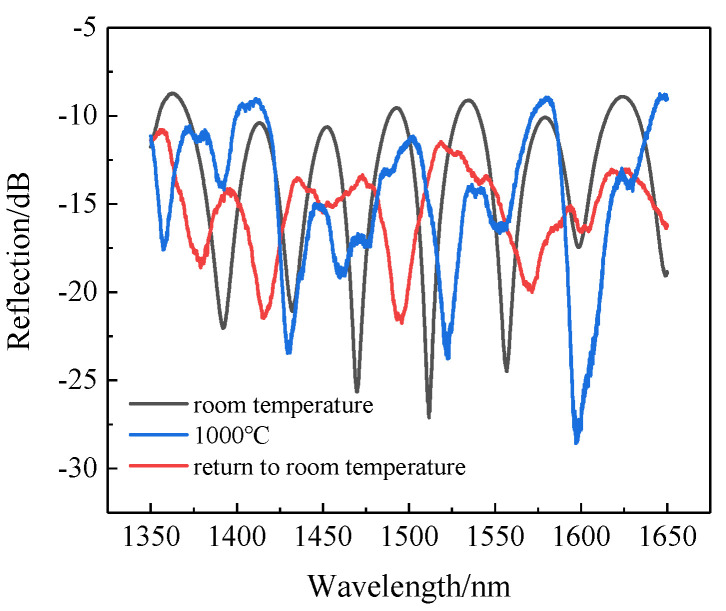
Changes in the spectra as the room temperature increased to 1000 °C.

Then, the temperature began to increase slowly from room temperature. When the temperature increased to 1000 °C, as shown in Figure 9, the sinusoidal spectra were distorted. When the temperature dropped from 1000 °C to room temperature, the distortion of the spectra could not be restored. After conducting several experiments, we found that the sensor did not suffer from spectral degradation at 900 °C. Therefore, in order to ensure that the sensor can work properly, the upper limit of the subsequent test temperature was set to 900 °C.

**Figure 10:**
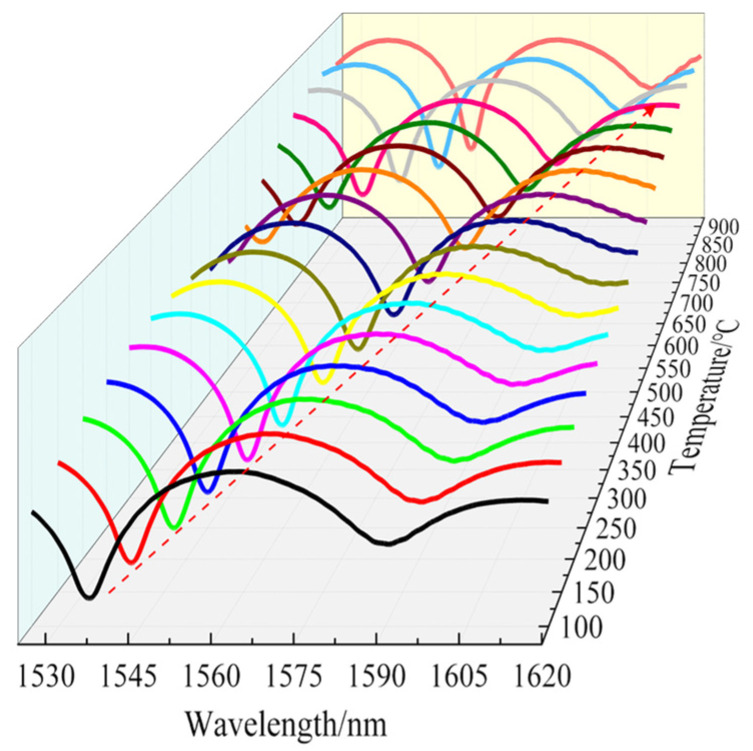
Temperature dependence of the spectrum.

Another sensor sample (L = 6 mm) was placed in the temperature furnace through a fixed quartz tube. The temperature was increased from room temperature to 900 °C at steps of 50 °C and cooled down to room temperature at the same step interval. Each temperature step was retained for about 30 min, which was done to equilibrate the spatial temperature distribution and avoid the effect of noise. We selected the dip near 1550 nm, which is commonly used in the communication band, and plotted the temperature dependence of the spectrum. Figure 10 shows that as the temperature increased from 100 °C to 900 °C, the wavelength of the interference fringe around 1542 nm showed a redshift. The wavelength dip shifted from 1542.305 nm to 1614.240 nm at 900 °C.

**Figure 11:**
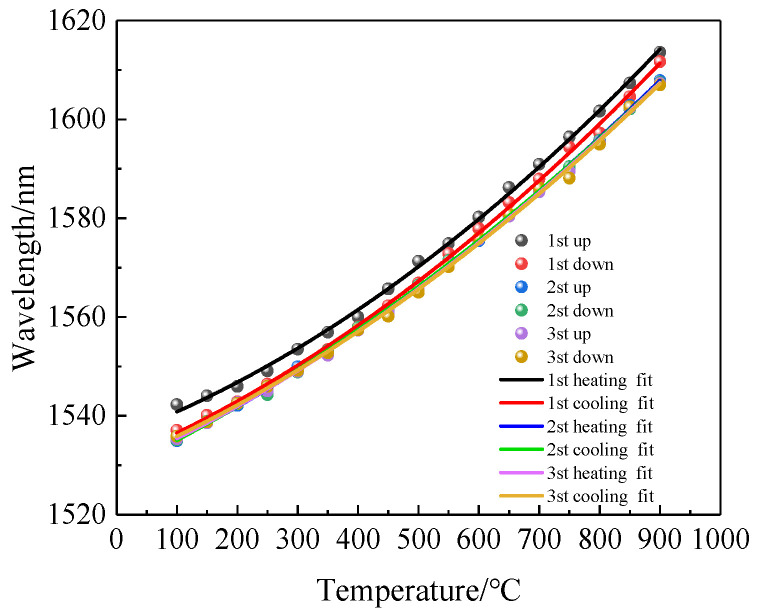
Spectra of the sensor after multiple high-temperature annealing cycles.

To ensure the rigor of this sensor’s temperature test, the heating and cooling cycles were repeated twice more. The results obtained by tracking and fitting the wavelengths to the same dip are shown in Figure 11. The response of the dip wavelength of the interference fringe showed a nonlinear characterization during the heating–cooling cycle process because the temperature coefficient is not a constant in a wide range (100~900 °C). We also observed that the dip wavelength of the first heating and cooling in Figure 11 did not coincide well with that of the second heating and cooling. It is clear that the sensor showed hysteresis. This is mainly due to the release of the internal stress of the fiber at a sufficiently high annealing temperature, which led to a small change in the refractive index of the fiber, so the spectrum changed to some extent. After two heating–cooling cycles, the fitting curves overlapped, and the fiber structure reached a stable state.

**Figure 12:**
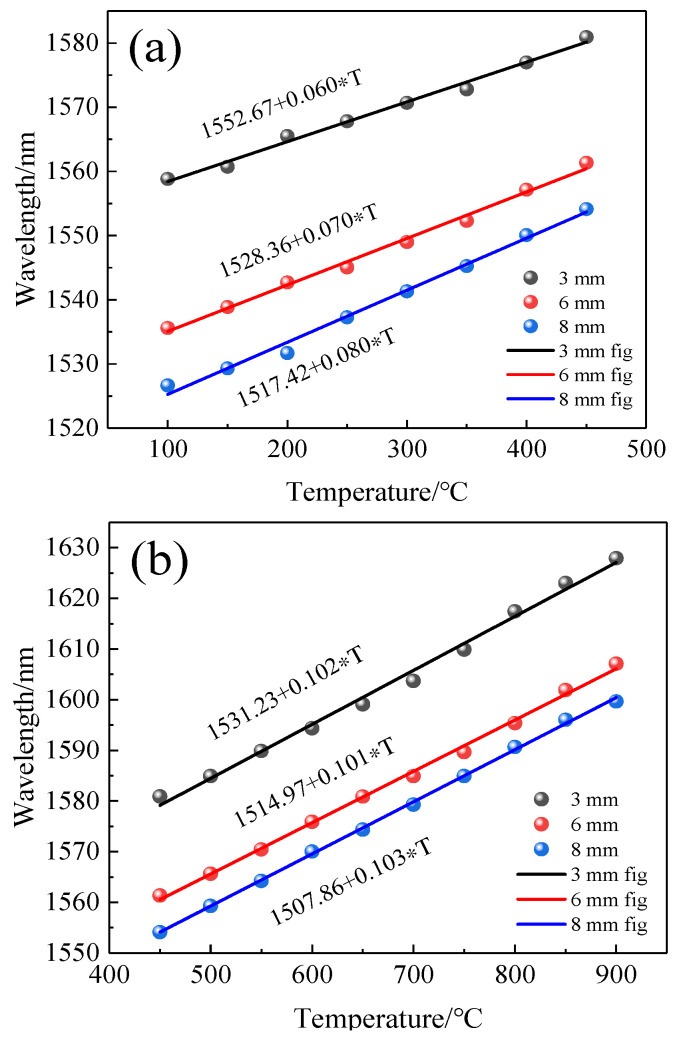
Temperature sensitivity of the MI structure with different lengths (L). (**a**) low-temperature region (100 °C~450 °C); (**b**) high-temperature region (450 °C~900 °C).

We prepared three sensors of different lengths (L = 3 mm, L = 6 mm, L = 8 mm) and after annealing them in the same way, they were placed in a temperature furnace and heated from room temperature to 900 °C in steps of 50 °C with a retention time of about 30 min for each temperature step. The spectra of the sensor with temperature for different lengths were recorded, and the dip near 1550 nm was chosen for the fit because the fitted curve of the sensor was nonlinear in the range of 100 °C to 900 °C as shown in Figure 11. Therefore, we divided the whole temperature range into two linear parts to obtain better linearity, i.e., a linear fit from 100 °C to 450 °C and 450 °C to 900 °C. The fitted curves are shown in Figure 12a,b. In the range of 100~450 °C, the sensitivity of three sensors with different lengths was 60 pm/°C, 70 pm/°C, and 80 pm/°C. In the range of 450~900 °C, the sensitivity was 102 pm/°C, 101 pm/°C, and 103 pm/°C.

When the ambient temperature changes, the sensitivity of the interference dip wavelength in the interference spectrum can be expressed as [36]:

(6) $$\frac{\partial \lambda }{\partial T}=\frac{2}{2m+1}\left[\Delta n_{\mathrm{eff}}\frac{\partial L}{\partial T}+L\left(\frac{\partial n_{\mathrm{eff}}^{\mathrm{co}}}{\partial T}-\frac{\partial n_{\mathrm{eff}}^{\mathrm{cl}}}{\partial T}\right)\right]$$

where T represents temperature. ∂L/∂T=αL represents the thermal expansion change of the fiber length with temperature change, where α represents the thermal expansion coefficient. We refer to the thermal expansion coefficient of SiO_2_ as $5.5\times 10^{-7}$. $\partial n_{\mathrm{eff}}^{\mathrm{co}}/\partial T=\xi_{\mathrm{eff}}^{\mathrm{co}}n_{\mathrm{eff}}^{\mathrm{co}},\partial n_{\mathrm{eff}}^{\mathrm{cl}}/\partial T=\xi_{\mathrm{eff}}^{\mathrm{cl}}n_{\mathrm{eff}}^{\mathrm{cl}}$ represents the change of the effective refractive index of the core mode and the cladding mode with the change of temperature, where $\xi_{\mathrm{eff}}^{\mathrm{co}}$ and $\xi_{\mathrm{eff}}^{\mathrm{cl}}$ represent the effective thermal light of the core mode and the cladding mode, respectively. Their values are approximately equal to $6.44\times 10^{-6}$ [37]. The sensitivity of the characteristic wavelength can be expressed as:

(7) $$\frac{\partial \lambda }{\partial T}=\frac{2L}{2m+1}\left(\Delta n_{\mathrm{eff}}\alpha +\xi_{\mathrm{eff}}^{\mathrm{co}}n_{\mathrm{eff}}^{\mathrm{co}}-\xi_{\mathrm{eff}}^{\mathrm{cl}}n_{\mathrm{eff}}^{\mathrm{cl}}\right)$$

According to Formula (7), the sensitivities of three sensors with different lengths (L = 3 mm, 6 mm, 8 mm) were calculated as 93 pm/°C, 106 pm/°C, and 112 pm/°C, respectively. The experimental results showed that the change of length had little effect on the sensitivity. It can be seen from Figure 8 that $\Delta n_{\mathrm{eff}}$ increased with the increase in the wavelength; for three sensors with different lengths, the interference valley was not the same wavelength, which is one of the reasons for the deviation between the experimental results and the calculated values, and the experimental environment and equipment will produce a little error in the results of the experimental field.

**Figure 13:**
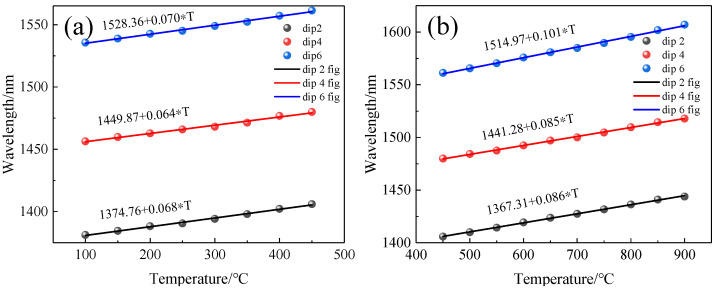
Temperature sensitivity of the MI structure with different dips. (**a**) low-temperature region (100 °C~450 °C); (**b**) high-temperature region (450 °C~900 °C).

We also observed the changes of dips at different wavelengths with temperature. We selected the 2nd, 4th, and 6th dips of the sensor with a 6 mm length as shown in Figure 4b and drew Figure 13a,b. By piecewise fitting the three dips, we can see that the 6th dip with the longest wavelength had the highest sensitivity, which was 70 pm/°C and 101 pm/°C, respectively, in two temperature ranges.

When the length of the sensor was 6 mm, we can calculate the sensitivities of the interference valleys (dip2, dip4, dip6) with different wavelengths according to Formula (7), which were 82 pm/°C, 87 pm/°C, and 106 pm/°C, respectively. This result is similar to the experimental results, and we conclude that the sensitivity of this sensor will increase with the increase in wavelength.

**Figure 14:**
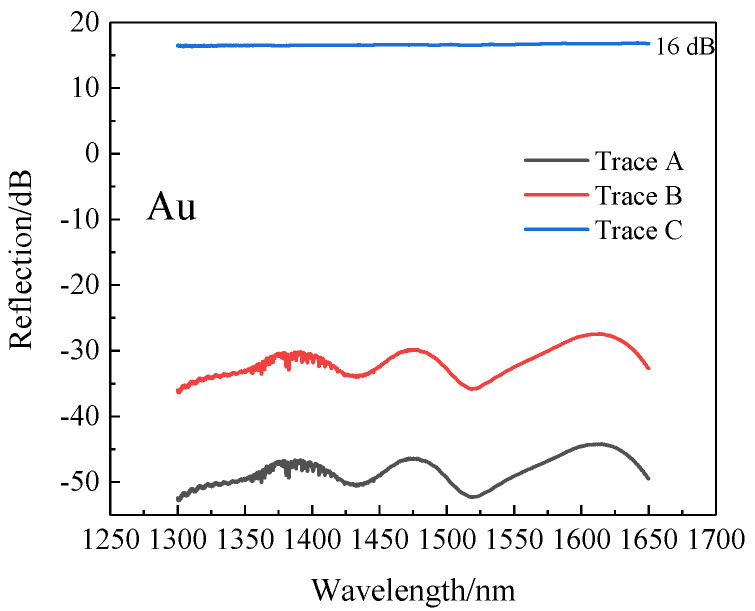
Original spectra with or without metal film. Trace A: without gold film, Trace B: with gold film, Trace C was calculated as Trace B minus Trace A.

The sensor fabricated in the experiment is based on Michelson interference and is a reflective type. The fiber has an end-face reflectivity of only 4%. In order to increase the intensity of the reflected light, we selected gold to deposit on the SMF ends by the vacuum evaporation coating method. The principle is that under high vacuum, using the principle of resistance evaporation, the evaporated material is heated at a large current on the evaporation boat, so that it is melted and evaporated at high temperature, and the required film is deposited on the sample. The reflection spectra of the SMFs with gold coating and without coating were saved and are plotted in Figure 14. The curve marked by Trace A is the original reflection spectrum of the gold-free fiber. Trace B is the reflection spectrum of the optical fiber coated with gold film. The ordinate value of Trace C was calculated by subtracting Trace A from Trace B. The reflection intensity of the gold-coated fiber was 16 dB higher than that of untreated fiber, so the reflectivity of the end face was greatly improved.

**Figure 15:**
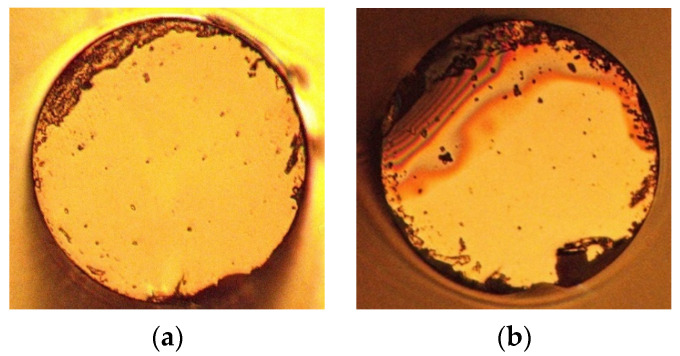
End face of the fiber with gold film under a microscope (**a**) Gold film before high-temperature annealing; (**b**) Gold film after high-temperature annealing.

The image of the end face with gold film of the sensor (L = 6 mm) under the microscope is shown in Figure 15a. Considering the melting point of gold is 1064 °C, we still set the upper limit temperature to 900 °C. The sensor was placed in the temperature furnace from room temperature to 900 °C and was then cooled to room temperature; this is an annealing process. Figure 15b shows the optical fiber end face microscope image after high-temperature annealing.

**Figure 16:**
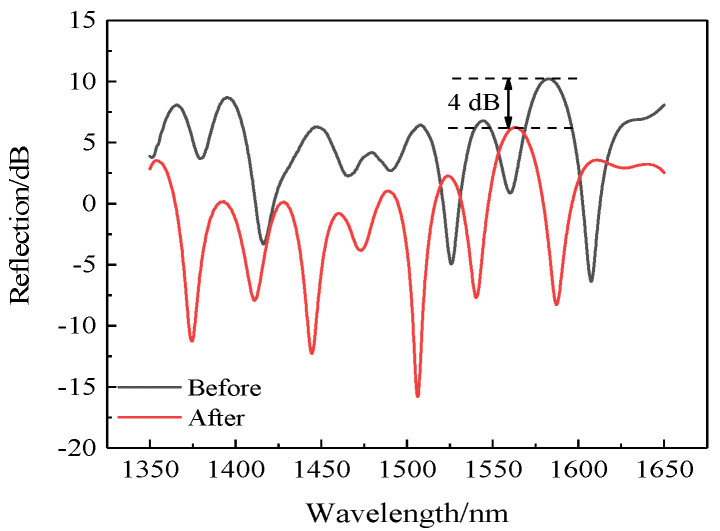
Spectra of the sensor before and after high-temperature annealing.

We recorded the spectral data before and after annealing, shown in Figure 16. Obviously, the spectra before and after high-temperature annealing also changed slightly, which was also due to the change in the internal stress of fiber caused by the high temperature. Although the reflected light intensity weakened, the overall intensity was still much higher than that of sensors without coating.

**Figure 17:**
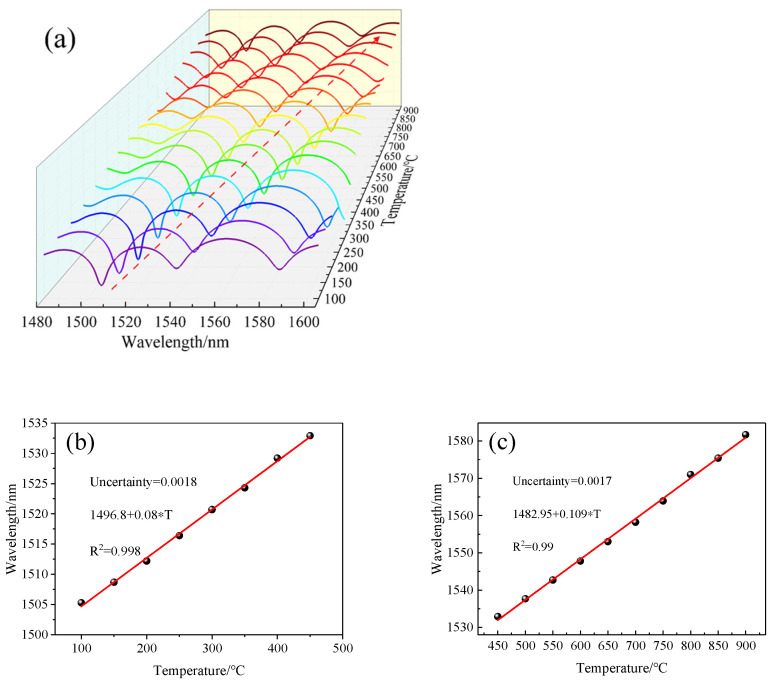
(**a**) Spectral changes with temperature; (**b**) Temperature sensitivity of the sensor structure by linear fitting at 100 °C~450 °C; (**c**) at 450 °C~900 °C.

**Figure 18:**
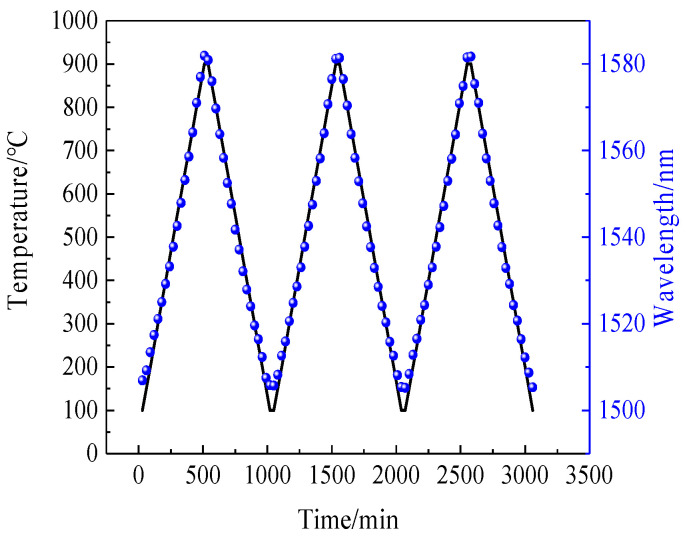
Three heating and cooling repeats of the 1506 nm dip.

We then put the annealed gold-coated sensor back into the temperature furnace; the temperature was increased from room temperature to 900 °C at intervals of 50 °C and then similarly cooled to room temperature. The retention time at each temperature step was about 30 min, and the spectrum was recorded. According to the data, the 1506-nm-wavelength dip was selected for analysis; the schematic of the spectral red shift shown in Figure 17a–c was obtained by piecewise fitting of the 1506 nm dip by Figure 18. Therefore, the temperature sensitivity of the sensor with golden film was obtained. From Figure 17b, it can be seen that the sensitivity of the sensor in the low-temperature region was 80 pm/°C; from Figure 17c, the sensitivity of the high-temperature region was 109 pm/°C. Moreover, the linear R^2^ in the low- and high-temperature ranges was 0.997 and 0.998, and the uncertainty was 0.0018 and 0.0017, respectively, which proves that the sensor had high linearity and low error. This means that gilding on the end face of the sensor can effectively increase the reflection while weakly affecting the temperature sensitivity.

We also repeated the process of heating the sensor from room temperature to 900 °C and then reduced it to room temperature three times; the recorded spectral data are shown in Figure 18. It can be seen intuitively from the diagram that the dip drift during the heating and cooling processes was basically the same for the three repeats.

**Figure 19:**
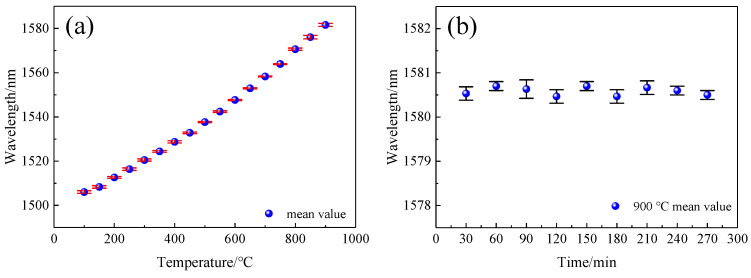
(**a**) Mean and standard deviation of the temperature dependence of the 1506 nm dip; (**b**) Mean and standard deviation of the 1506 nm dip at 900 °C.

According to Figure 18, Figure 19a was drawn by calculating the mean and standard deviation of the dip drift with temperature during the heating and cooling processes. The standard deviation was minor, indicating that the repeatability of the sensor was excellent. Finally, the sensor was kept at 900 °C for 270 min, and this was repeated three times. As shown in Figure 19b, the dip drift was always maintained in a small range at 900 °C, and for a long time, did not exceed ± 0.3 nm, meaning that the measurement error at 900 °C was less than that at 3 °C. This also shows that the sensor had good stability at that temperature.

Many scholars have made contributions to the research of fiber temperature sensors. Table 2 summarizes the sensitivity, measurement range, and fabrication methods of the fiber optic temperature sensors reported in this work and previous literature. In this paper, we use a simple fabrication method to achieve the measurement of 100 °C to 900 °C with an SMF optic sensor.

## 4. Conclusions

An optical fiber probe sensor with a simple structure was fabricated in an SMF by an optical fiber splicing machine. The temperature sensitivity, repeatability, and stability at high temperature were studied. The maximum measurement temperature reached 900 °C, and the sensor had good stability in a high-temperature environment. A gold film was coated on the end of the sensor to increase the reflectivity. The sensitivities of the sensor were 80 pm/°C and 109 pm/°C in the range of 100~450 °C and 450~900 °C, respectively. The high-temperature sensor has the advantages of simple manufacturing, a simple structure, low cost, and high sensitivity, so the sensor is particularly suitable for applications in industry, mining, and chemistry.

## Figures and Tables

**Table 1:** Relationships between the mode and cut-off frequency.

LP Mode	$U_{0}$	$U_{\infty }$
${\mathrm{LP}}_{01}$	0	2.40483
${\mathrm{LP}}_{11}$	2.40483	3.83171
${\mathrm{LP}}_{21}$	3.83171	5.13562
${\mathrm{LP}}_{12}$	5.52008	7.01559
${\mathrm{LP}}_{22}$	7.01559	8.41724

**Table 2:** Comparison of the fiber temperature sensors.

Authors	Methods	Range	Sensitivity
Present work	SMF	100~900 °C	109 pm/°C
Wang et al. [13]	FBG	0~350 °C	20.1 pm/°C
Zhou et al. [15]	LPFG	Room temperature~875 °C	0.37 nm/°C
Zhao et al. [32]	TCF	30~800 °C	0.14 nm/°C
Zhao et al. [33]	TCF and SHF	30~100 °C	10.37 pm/°C
Han et al. [34]	SMF	100~900 °C	10.87 pm/°C
